# WCST Performance in Schizophrenia and Severe Depression with Psychotic Features

**DOI:** 10.5402/2012/373748

**Published:** 2012-01-23

**Authors:** Ahmed Rady, Adel Elsheshai, Heba Abou el Wafa, Osama Elkholy

**Affiliations:** Department of Psychiatry, Alexandria University, P.O. Box 518, Alexandria 21511, Egypt

## Abstract

*Background*. Differentiating between schizophrenia and major depression with psychotic features often reveals diagnostic dilemma. Both share psychotic features and severe impairment in occupational functions. Severe psychomotor retardation, not uncommon in psychotic depression, may simulate negative symptoms of schizophrenia. Our work aims at utilizing Wisconsin Card Sorting Test (WCST) performance as a potential differentiating neurocognitive tool. *Subjects and Methods*. 60 patients were recruited randomly from the outpatient service at Alexandria University Hospital: 30 patients with schizophrenia and 30 patients with chronic psychotic depression. They were subjected to Clinical Global Impression for Severity (CGI-S) scale and Wisconsin Card Sorting Test (WCST) 128 card computerized version. *Results*. Both groups were balanced in terms of gender distribution, severity and duration of illness. The study compared all parameters of WCST. Only perseverative errors showed mild significant difference (*P* < 0.05) that disappeared when applying Bonferroni adaptation, setting significance level at 0.01 instead of 0.05. *Conclusion*. Performance on WCST is similar in schizophrenia and severe depression with psychotic features in most of the measured parameters and hence could not serve as a supplementary tool differentiating between both diagnoses in our study.

## 1. Introduction

The prevalence of major depression in primary care practice is 4.8% to 9.2% rendering mood disorders to be the most important psychiatric illness in primary care settings [1]. Though some psychiatrists believe that psychotic depression is uncommon, studies continue to demonstrate that 16% to 54% of depressed patients have psychotic symptoms; delusions occur without hallucinations in about half to two thirds in adults with psychotic depression, while hallucinations are unaccompanied by delusions in 3% to 25% of those patients [1].

Major depression with psychotic features seems to present a distinct disorder from major depression without psychotic features; the presence of psychosis, independent of depression or level of general psychopathology, is predictive of response to antidepressants monotherapy, supporting such distinction [2]. The continuous performance test has been used to demonstrate deficits in core attentional functioning in schizophrenia, mania, and major depression [3–6]. Some authors have demonstrated attentional performance to be poor particularly in psychotic depression and schizophrenia rather than in depression lacking psychosis, providing another asset to strengthen the similarity of psychotic depression with schizophrenia rather than nonpsychotic depression [7]. Deficits in sustained attention in continuous performance test are shown to represent stable vulnerability indicators for schizophrenia and state-dependant indicator for major depression [8].

The Wisconsin Card Sorting Test (WCST), a widely used neuropsychological index of prefrontal cortical function, demonstrated depressed patients to have significant deficits on multiple WCST measures compared to healthy individuals. These deficits were correlated with the severity of depression and were less severe than those demonstrated by patients with schizophrenia, providing neuropsychological evidence for significant prefrontal cortical dysfunction in depression [9–11].

Though WCST has been utilized to assess frontal lobe functions in schizophrenia and depression, specific emphasis on WCST performance in major depression with psychotic features subtype is lacking. Our work attempts to utilize WCST as potential differentiating tool between schizophrenia and psychotic depression.

## 2. Subjects and Methods

60 patients aged between 18 and 50 yrs were randomly recruited from the outpatient psychiatric service of Alexandria University Hospital. The study was approved by the ethics committee of Alexandria Faculty of Medicine and patients signed a written consent form. Diagnosis was made by structured clinical interview (SCID I for DSM IV) in conformity with criteria of DSM IV (Diagnostic and Statistical Manual of Mental Illness in its 4th edition) [12, 13]. Only patients scoring 4 or more on the Clinical Global Impression for Severity CGI-S scale [14] were included so that only severe cases were recruited.

To minimize the effect of medications on WCST performance, only patients who were not taking their medication for at least 1 week before presenting or being brought by a family member to the outpatient service were included. All subjects are off treatment for at least one week before recruitment. Patients having chronic debilitating diseases, mental retardation, and handicap rendering assessment unreliable were excluded.

Subjects included 30 patients with schizophrenia and 30 patients with major depression with psychotic features chronic course. All were rated on the Brief Psychiatric Rating Scale (BPRS) [15], a valid and reliable questionnaire to assess some demographic data as well as duration of illness and WCST computerized version 128 card produced by Psychological Assessment Resources PAR Inc. USA [16]. Diverse measures on the WCST were assessed including number of administered trials, completed sets, percentage of correct answers and errors, percentage of perseverative errors, number of trials to achieve the first completed category, and number of failed categories [16, 17].

### 2.1. Statistical Methods

Data were analyzed using PC with Statistical Package for Social Sciences SPSS version 13; the 0.05 was used as cutoff value for statistical significance. To assess comparison between schizophrenic and depressed patients regarding the whole set of parameters measured by the WCST the Bonferroni correction could be applied by setting the level of significance at 0.01, instead of 0.05, for each WCST parameter assessed. Parametric testing was used to compare between independent groups as regards means and frequencies for various parameters.

## 3. Results

Both schizophrenic and psychotically depressed patients who participated in our study showed no statistical difference in terms of gender distribution, age, duration of illness, severity as assessed by CGI-S, and finally BPRS as a general psychometric tool commonly applied to mental illness (Table 1).

As regards the different parameters assessed by the WCST, no statistical difference could be found apart from percentage of perseverative errors which was the only parameter to show statistical difference (*P* < 0.05) (Table 2).

The application of Bonferroni adaptation to the significance level for the whole set of items included in the WCST eliminates such difference (Table 3).

## 4. Discussion

The WCST is one of the most widely used psychological testing tools to assess executive functioning such as problem solving, decision making, inhibitory control, and working memory. WCST performance was consistently found to be lower in patients with schizophrenia; however, it does not seem to be specific to that disorder, because schizophrenia is characterized by a broad base of cognitive impairment, with varying degrees of deficit in all ability domains, as measured by standard clinical tests. Since the WCST assesses various functions, it is difficult to differentiate its part in working memory from problem-solving capacity or other executive functions. Therefore, it is not surprising that patients with unipolar depression performed poorly on the WCST. In one systemic review, 14 out of 15 studies demonstrated impairments of executive functioning in major depression [18]. Several brain regions in addition to the prefrontal cortex were shown to affect performance on the WCST. The disturbances in prefrontal areas that were demonstrated may be a necessary but not sufficient condition for a poor WCST performance [18].

Several studies revealed contradictory results. Authors found that the performance of patients with chronic schizophrenia improved on the WCST when they received explicit card-by-card instructions. However, performance dropped to baseline levels when the instructions were withdrawn. They concluded that patients with schizophrenia were unable to learn the WCST, suggesting unremediable deficits that were probably linked to a prefrontal dysfunction; they also proposed that their failure did not result from not knowing but from not doing; in other words, the necessary information was received but was not used to change behaviour [19]. The patients were able to learn to perform other, nonprefrontal tasks, suggesting that the performance deficit on the WCST was not due to inattention or lack of effort. Such improvement in WCST performance in schizophrenic patients has been also shown in recent studies [20–22].

To integrate these notions, one study proposed a model specifically designed to explain positive psychotic symptoms. The model shows a failure in acute schizophrenia to integrate stored memories of past regularities of perceptual input with ongoing motor programs [23]. Some authors reported that there was no evidence of transfer of training effects across problem-solving tests, despite the similarity in the cognitive demands imposed by the instruments patients tested on [24].

Some authors found improvements after monetary reinforcements [25]; others found greater improvement after instructions without monetary reinforcement [26]. Studies reported by Bellack et al. revealed that performance improved on the WCST when they combined monetary reinforcement with detailed instructions [24, 27]. These findings indicate that some patients with schizophrenia may be able to learn the WCST, suggesting that their “frontal lobe” deficits are remediable. Although the “frontal lobe” hypothesis of schizophrenia has a venerable history, we have seen that there are still gaps in our understanding of the precise nature of the deficit involved, as well as the reversibility of this deficit through reinforcement.

Though many authors tackled the poor WCST performance in schizophrenia and depressed patients with potential clinical applications for that but literature evaluating WCST in the subtype of psychotic depression is scarce and whether WCST can serve as a potential differentiation tool aiding diagnosis was not evoked in the literature.

## 5. Limitations of the Study

Our study is limited by small sample so that extrapolation and generalization of the results are difficult and a definite need for replication on larger numbers to support our findings. Although there were high numerical differences between the 2 groups, they were not significant, probably due to the low power/low sample size. Another weak point is attributed to the fact that patients were not drug naïve even if they were off medication for at least 1 week when presenting to the outpatient service, spontaneously or with a family member, before recruitment. Replication on newly diagnosed drug-naïve patients will be of great added value.

## 6. Conclusion

Schizophrenic and depressed patients show poor performance on WCST. In our study, WCST could not serve as additional tool helping in differential diagnosis. Due to limitations of our study, more studies are needed on larger samples and medication naïve patients to explore this area.

## Figures and Tables

**Table 1:** Demographic data, duration, and severity of illness in schizophrenic and psychotically depressed patients (no difference found).

	Patients with schizophrenia (*n* = 30)	Patients with major depression with psychotic features (*n* = 30)	Statistic test
Gender	Female Male	Female Male	
	40% 60%	50% 50%	*χ* ^2^ = 0.53
Age (yrs)	35.2 ± 8.01	36.9 ± 7.98	*U* = 0.52
Illness duration (yrs)	3.85 ± 2.01	5.1 ± 2.83	*U* = 3.13
CGI-S score	5.1 ± 0.55	4.6 ± 0.5	*U* = 3.57
BPRS score	49.45 ± 3.05	47.85 ± 4.07	*U* = 1.72

**Table 2:** WCST parameters in schizophrenic and psychotic depression patients (significance level set at *P* < 0.05).

WCST parameter	Patients with schizophrenia (*n* = 30)	Patients with major depression with psychotic features (*n* = 30)	Statistic *U* bitailed
Number of trials	118.4 ± 19.7	128 ± 0	2.67 (*P* > 0.05)
% of Errors	47.2% ± 10.79	34% ± 15.2	1.04 (*P* > 0.05)
% of perseverative errors	30% ± 11.4	18% ± 9.97	0.01* (*P* < 0.03)
Number of completed categories	3 ± 1.3	3.8 ± 1.64	2.09 (*P* > 0.05)
Number of trials to the 1st completed category	18.2 ± 12.08	14.4 ± 5.09	1.5 (*P* > 0.05)

*P < 0.05.

Note. Better performance on WCST is indicated by *larger* number of trials but *less* percentage of errors, number of completed categories, and number of trials to the 1st completed category.

**Table 3:** WCST parameters in schizophrenic and psychotic depression patients (with Bonferroni adaptation applied to statistics and significance level set at *P* < 0.01). No statistical difference shows up.

WCST parameter	Patients with schizophrenia (*n* = 30)	Patients with major depression with psychotic features (*n* = 30)	Statistic *U* bitailed
Number of trials	118.4 ± 19.7	128 ± 0	2.67 (*P* > 0.01)
% of Errors	47.2% ± 10.79	34% ± 15.2	1.04 (*P* > 0.01)
% of perseverative errors	30% ± 11.4	18% ± 9.97	0.01 (*P* > 0.01)
Number of completed categories	3 ± 1.3	3.8 ± 1.64	2.09 (*P* > 0.01)
Number of trials to 1st completed category	18.2 ± 12.08	14.4 ± 5.09	1.5 (*P* > 0.01)

*P < 0.01.

By applying Bonferroni correction with significance level set at (*α*/*n* = 0.05/5), that is, 0.01 instead of 0.05 for each parameter in the whole set, no difference shows up between schizophrenic and psychotically depressed patients regarding the whole set of parameters measured by WCST.
